# Pattern of Maternal Mortality in a Tertiary Care Hospital of Patna, Bihar

**DOI:** 10.4103/0970-0218.45381

**Published:** 2009-01

**Authors:** Rashmi Singh, Nivedita Sinha, Krishnadas Bhattacharyya, Rama Ram

**Affiliations:** Department of Community Medicine, Patna Medical College and Hospital, Patna, India; 1 Department of Community Medicine, Calcutta National Medical College, Kolkata, India. E-mail: krishnadas_001@rediffmail.com; 2 Department of Community Medicine, MGM Medical College, Kishangaunge, Bihar, India

Sir,

In India, women of child-bearing age constitute approximately 19% of the population.(1) Major portions of these women posses factors that are conducive to increased maternal mortality. Bihar along with UP, MP, Rajasthan, and Punjab have a maternal mortality rate (MMR) over 450 per 100,000 live births.(2) This study was undertaken to find out the root causes of such deaths.

During the study period of 2 years from August 2003 to July 2005 in three settings of a Medical College Hospital, all the women who died due to complications of pregnancy and childbirth within 42 days of delivery were selected. The relevant facts and history were noted from case records, attendants, and missing information was collected later by home visits. Data on a total of 329 maternal deaths were recorded. The total number of live births was 8422 during the study period.

In the first and second years of the study, maternal deaths were 180 and 149, respectively. The MMR was 4159.93 and 3638.58 per 100,000 live births, respectively making the overall MMR 3906.44/100,000 live births. Among 329 total maternal deaths, the maximum were in the age group of 21 to 30 years old (56.54%) followed by 31 to 40 years old (22.49%). The death rate for mothers from rural areas was higher (67.17%). Maternal deaths were observed more among illiterate women (57.75%). The highest number of maternal deaths occurred among those who were second or third gravida (48.94%) followed by fourth gravida and above (26.14%) [Table 1]. A total of 85.11% of maternal deaths happened among those who had not received adequate antenatal care services. Toxemia of pregnancy (24.01%) was observed as the major cause of maternal death followed by sepsis (17.93%) [Table 2]. Anemia accounted for 15.81% of maternal deaths. In the majority of cases (58.36%), the women went to the hospital more than 24 hours to less than 1 week after the onset of complications and 12.16% of the women went to the hospital more than 1 week after the onset of complications.

**Table 1:** Gravida, utilization of maternal care services, and maternal mortality

Characteristics	Number of maternal deaths (n = 329)	Percentage
Gravida		
Primi	82	24.92
2^nd^ and 3^rd^	161	48.94
4^th^ and above	86	26.14
Antenatal care		
Adequate (≥ 3)	49	14.89
Inadequate (< 3)	280	85.11

**Table 2:** Causes of maternal deaths

Causes	Number	Percentage
Anemia	52	15.81
Hemorrhage	53	16.11
Toxemia	79	24.01
Sepsis	59	17.93
Other direct causes	28	8.52
Other indirect causes	58	17.83

This study has shown a higher maternal mortality rate (3906.44/100,000 live births) and may be due to being a tertiary care hospital based study, where more complicated cases are admitted. The majority of deaths were in the age group of 21 to 30 years old (56.54%). Baul, *et al*.(3) had a similar observation of 51.8%. Women from rural areas contributed to a major share of maternal mortality (67.17%). Chi, *et al.*(4) also reported higher maternal mortality among women of rural areas. The highest rate of deaths was among illiterate women. This is in conformity with Rajaraman, *et al.*(5) The majority of deaths were among 2^nd^ and 3^rd^ gravida (48.94%) and among those who had not received adequate antenatal care services (84.11%). Baul, *et al.*(3) and Rajaraman, *et al.*(5) had similar observations. The direct obstetric causes accounted for 66.56% of maternal deaths; toxemia (24.01%), sepsis (17.93%), and hemorrhage (16.11%) were the major causes. Anemia accounted for another 15.81% of deaths. These findings are in conformity with the findings of Rajaraman, *et al.*(5) and Ramteke, *et al.*(6)

This study has found the root causes of very high maternal mortality rate to be illiteracy, inadequate antenatal care services, and a delay in the initiation of treatment.
